# Glutathione S-Transferase Regulation in *Calanus finmarchicus* Feeding on the Toxic Dinoflagellate *Alexandrium fundyense*

**DOI:** 10.1371/journal.pone.0159563

**Published:** 2016-07-18

**Authors:** Vittoria Roncalli, Michelle J. Jungbluth, Petra H. Lenz

**Affiliations:** 1 Békésy Laboratory of Neurobiology, Pacific Biosciences Research Center, University of Hawai‘i at Mānoa, 1993 East-West Road, Honolulu, HI 96822, United States of America; 2 Department of Oceanography, 1000 Pope Rd., University of Hawai‘i at Mānoa, Honolulu, HI 96822, United States of America; Stazione Zoologica Anton Dohrn, Naples, ITALY

## Abstract

The effect of the dinoflagellate, *Alexandrium fundyense*, on relative expression of glutathione S-transferase (GST) transcripts was examined in the copepod *Calanus finmarchicus*. Adult females were fed for 5-days on one of three experimental diets: control (100% *Rhodomonas* spp.), low dose of *A*. *fundyense* (25% by volume, 75% *Rhodomonas* spp.), and high dose (100% *A*. *fundyense*). Relative expression of three GST genes was measured using RT-qPCR on days 0.5, 1, 2 and 5 in two independent experiments. Differential regulation was found for the Delta and the Sigma GSTs between 0.5 to 2 days, but not on day 5 in both experiments. The third GST, a microsomal, was not differentially expressed in either treatment or day. RT-qPCR results from the two experiments were similar, even though experimental females were collected from the Gulf of Maine on different dates and their reproductive output differed. In the second experiment, expression of 39 GSTs was determined on days 2 and 5 using RNA-Seq. Global gene expression analyses agreed with the RT-qPCR results. Furthermore, the RNA-Seq measurements indicated that only four GSTs were differentially expressed under the experimental conditions, and the response was small in amplitude. In summary, the *A*. *fundyense* diet led to a rapid and transient response in *C*. *finmarchicus* in three cytosolic GSTs, while a fourth GST (Omega I) was significantly up-regulated on day 5. Although there was some regulation of GSTs in response the toxic dinoflagellate, the tolerance to *A*. *fundyense* by *C*. *finmarchicus* is not dependent on the long-term up-regulation of specific GSTs.

## Introduction

The glutathione S-transferases (GSTs) belong to a large family of proteins involved in detoxification [1–3]. Thus, GSTs are often targeted as biomarkers in ecotoxicology. This target gene approach has been used to study the effect of toxins on the copepods in the genus *Calanus*, including *C*. *finmarchicus*, *C*. *glacialis*, *C*. *helgolandicus* and *C*. *sinicus* by measuring relative gene expression of target GSTs using real-time quantitative polymerase chain reaction (RT-qPCR) [4–12]. These studies have usually focused on single GSTs, such as a microsomal GST [4–6] or a member of the cytosolic Delta GST [7–12]. However, the GST family is characterized by multiple gene duplications in eukaryotes, exceeding 30 genes in many insects [13]. Furthermore, although many GSTs are involved in cellular detoxification, responses of individual genes differ depending on the stressor [14,15]. Thus, with the target gene approach, the use of a single GST as a detoxification marker, may not be sufficient to characterize the response in *C*. *finmarchicus* or its congeners.

In *C*. *finmarchicus*, the diversity of the GST family exceeds that of many insects [16]. A total of 41 different GST unigenes have been predicted for *C*. *finmarchicus* from two independently generated transcriptomes [16]. These included multiple members of the cytosolic, microsomal and mitochondrial classes [16]. The largest number of genes (32 genes) was predicted for the cytosolic class, and many of these appeared to be from recent duplication events [16]. Cytosolic GSTs are activated in response to oxidative damage and/or exposure to toxins including pesticides [2], although they are also involved in other cellular functions [3]. Like many calanoid copepods, *C*. *finmarchicus* has a high tolerance for many toxic phytoplankton species, including the saxitoxin-producing dinoflagellate, *Alexandrium fundyense* [17,18]. Thus, detoxification through the activation of one or more GST could contribute to the copepod’s ability to consume the dinoflagellate with little effect on its survival [18].

In the Gulf of Maine, zooplankton biomass in the spring and summer is dominated by the calanoid copepod *C*. *finmarchicus* [19,20]. The copepod is a key food source for many economically important fishes such as larval herring and mackerel [21]. During summer, *C*. *finmarchicus* co-occurs with blooms of *A*. *fundyense*. These blooms occur annually with dinoflagellate cell densities often above 10 cells mL^−1^,and local blooms reaching 100 cells mL^−1^ [22,23]. Although copepod survival is high, reproductive success is reduced [18,24]. Furthermore, *C*. *finmarchicus* responds with a generalized cellular stress response after two days on a dinoflagellate diet [24]. Surprisingly, detoxification did not appear to be a major component of the physiological response [24], raising questions whether the response had been missed due to a limited number of time points, and whether the result was reproducible.

Therefore, in this study, relative expression of GSTs in *C*. *finmarchicus* in response to a diet containing *A*. *fundyense* was investigated using RT-qPCR in two separate experiments using field-collected individuals. Adult females were fed on one of three diets: control, low dose (25% *A*. *fundyense* by volume) and high dose (100%) of *A*. *fundyense* for five days. Change in relative expression of three target GSTs and three reference genes was measured in adult females from two separate experiments at four different time points (0.5 to 5 days). In addition, RT-qPCR results were compared with high-throughput sequencing (RNA-Seq) results in one experiment for two time points (2 and 5 days). RNA-Seq was also used to measure relative expression of 36 additional GSTs.

## Materials and Methods

### Field collection, phytoplankton cultures and maintenance of *Calanus finmarchicus*

*Calanus finmarchicus* were collected using a vertical net tow (75 cm diameter, 560 μm mesh) in June (14^th^) and July (1^st^) of 2012 in the Gulf of Maine near Mount Desert Rock (Lat: 44° 2’N; Long: 68°3’W, USA). No specific permissions were required for these collections. Copepods were diluted in surface seawater and kept overnight at 10°C without food prior to the experiment. The summer of 2012 was described as a moderate bloom year with detectable levels of *A*. *fundyense* (50–100 cells L^−1^) being reported on June 1 (http://www.whoi.edu/website/northeast-psp/).

The phytoplankton cultures used in this study were used also in two 2 parallel studies where more detailed description are available in two other publications [18,21]. Briefly, the toxic dinoflagellate *A*. *fundyense* (clone GTCA28, origin: western Gulf of Maine, isolated in 1985) cultures were grown at 15°C on a 14:10 h light:dark cycle and then transferred to 10°C during mid- exponential growth for temperature equilibration prior to shipment from Woods Hole (MA) to Mount Desert island (ME) [18]. For the experiments, the cultures were maintained at the same light:dark cycle and 10°C, and diluted by 50% every two days with f/2-Si medium [18,24]. The flagellate *Rhodomonas* sp. clone (CCMP739) was maintained at 15–16°C in ambient outside natural light and diluted by 50% with f/2 medium after the third day [16,19]. The toxicity of *A*. *fundyense* was measured daily during the experiments as described in Roncalli et al., [18].

### Experimental design

Adult *Calanus finmarchicus* females were fed for 5 days on of three experimental diets: control, low dose of *A*. *fundyense* (LD) and high dose of *A*. *fundyense* (HD). In the control group, the non-toxic flagellate *Rhodomonas* sp. was added daily at 8000 cells mL^−1^d^−1^. Copepods in the LD group received daily rations of 50 cells mL^−1^d^−1^*A*. *fundyense* and 6000 cells mL^−1^d^−1^ *Rhodomonas* sp. (25:75 proportion by algal volume). Copepods in the HD group, were fed a diet of 100% of *A*. *fundyense* at daily rations of 200 cells mL^−1^d^−1^. The three experimental food suspensions had similar carbon content ranging between 304 and 358 μgC L^−1^ [18]. Three separate sets of biological replicates were set up for each treatment (control, LD and HD) and sampling times (0.5, 1, 2 and 5 days). Eight females were transferred into containers with 800 mL of seawater for RT-qPCR, and 15 females were transferred into 1500 ml containers for RNA-Seq. During the incubation period food rations were added daily, and each container was checked for dead individuals. The containers were kept in a Percival Model I-36VL Incubator System (Percival Scientific, Inc., Perry, IA, USA) at 10°C on a 14:10 hr light dark cycle.

For each sampling point (0.5, 1, 2 and 5 days), 4 adult females were removed from each treatment and biological replicate with separate experimental containers set up for the 0.5 and 1 day samples, and the 2 and 5 day samples. Harvested individuals were immediately transferred to 0.5 mL RNA later (Ambion), and stored at -80°C until RNA extraction. The first experiment was completed in June (15^th^ to 19^th^, 2012), and a second one in July (2^nd^ to 6^th^, 2012). In the July experiment, samples were not collected at the day 1 time point, however, females were harvested from the 1500 mL jars on days 2 and 5 during the July experiment and prepared for RNA-Seq [24].

### Gene expression using RT-qPCR

#### RNA extraction, cDNA synthesis and primer design

Total RNA was extracted from whole *C*. *finmarchicus* adult females (n = 1) using the QIAGEN RNeasy Mini Kit (QIAGEN Inc., Valencia, CA, USA), in conjunction with a Qiashredder column (QIAGEN Inc.), following the instructions of the manufacturer, with a final elution volume of 30 μL. RNA concentration and quality were checked using an Agilent Model 2100 Bioanalyzer (Agilent Technologies, Inc., Santa Clara, CA, USA). First-strand synthesis was performed using the QuantiTect Reverse Transcription Kit (Qiagen, Valencia, CA, USA), which is optimized for RT-qPCR down-stream applications. 1 μg of total RNA was reverse transcribed to cDNA, following the manufacturer’s instructions. The resulting cDNA was used as template for conventional PCR and RT-qPCR.

Relative expression was measured for three target GSTs (Calfi-Delta I, Calfi-Sigma VI and Calfi-mGST3 III) and three candidate reference genes (actin, 16S and elongation factor 1α) from a total of 36 cDNA libraries (3 replicates × 3 treatments × 4 time points). Primers were designed based on sequences obtained from the *C*. *finmarchicus de novo* assembly [25] using the primer design tools in Primer3 within the software Geneious (V6.1). Specific primers were designed to target the conserved domain for each gene of interest that was identified using the online program SMART (http://smart.embl-heidelberg.de) [26]. For all genes the amplicon size was less than 170 bp. The list of genes, forward and reverse primers, amplicon lengths and oligo efficiencies (E) are listed in Table 1. Secondary structure and primer accuracy were evaluated using the OligoAnalyzer Tool available in IDT (http://www.idtdna.com) and *in silico* PCR (Bioinfx http://bioinfx.net/cgi-bin/pcr). Each primer set was first tested with traditional PCR to optimize the temperature range (55–63°C) and primer concentrations (100–400 nM). PCR amplifications were performed in 25 μL reaction volumes using 2.5 μL 10× PCR Buffer minus Mg^2+^, 0.75 mM MgCl_2_, 0.75 μM of each primer, 0.5 mM of each dNTP, Invitrogen Taq polymerase (recombinant) at 0.25 units μL^−1^, 3 μL of template DNA and 16.5 μL of deionized water. Reaction conditions included denaturing at 95°C for 30 s, followed by 40 cycles of denaturation at 95°C for 30 s, the primer specific annealing temperature (between 55–58°C) for 30 s, extension at 72°C for 1 min, then the final extension step at 72°C for 4 min. Only primers that generated a single strong band on a 1.5% agarose gel with no bands visible in the no-template control (NTC) were further considered for the RT-qPCR analysis.

**Table 1:** RT-qPCR Primer Pairs.

Gene name*	Primer Direction	Primer sequence 5’-3’	L (bp)	E (%)
*GST genes*				
Calfi-Delta-I	F	TCAGGTCACCATCCACAAGC	143	100
	R	AGCAGTCCACATGGCTTTGA		
Calfi-Sigma-VI	F	CCCCTCCCCAGTAGAGCATA	167	98
	R	CTTCAACCTGAGAGCCCGAG		
Calfi- mGST-3-III	F	TCTTGCTCCCTGCTCAGAAT	120	99
	R	TTGCGGGCTCTTTGTTAAGT		
*Reference genes*				
EFA	F	AATATGGGCGGTGTGACAAT	127	100
	R	CTCCGACTCCAAGAACAAGC		
16S	F	CGTCTCTTCTAAGCTCCTGCAC	114	98
	R	AAGCTCCTCTAGGGATAACAGC		
actin	F	CCCAAGCCTATTGAGGTTCA	124	100
	R	CATACTGGGCCTTGGTGTGG		

List of genes of interest (Target genes, Reference Genes) used to measure gene expression in RT-qPCR. For each gene, primer sequence (f = forward, r = reverse), amplicon length (L) and PCR efficiency (%) are listed.

* Gene accession numbers; Calfi-Delta I GAXK01204953, Calfi-Sigma VI GAXK01204959, Calfi-mGST-3 III GAXK01204955, EFA GAXK01169633, 16S GAXK01168561, actin GAXK01020331.

#### Reverse-Transcription-Quantitative Real Time Polymerase Chain Reaction (RT-qPCR)

RT-qPCR experiments were performed to measure relative expression of the three target and three reference genes using the cDNA previously generated (see above). These experiments were performed on a LightCycler 96 System (Roche) thermal cycler in a final volume of 25 μL containing the following: 12.5 μL of Fast Start SYBR Green Master Mix (Roche), 2 μL of cDNA template and 1 μL of each oligo (final concentration 400 nM). The RT-qPCR thermal profile included pre-incubation at 95°C for 10 min followed by 50 cycles of 95°C for 30 s, primer specific annealing temperature for 1 min and 72°C for 30 s. Melt curve analyses were checked for the presence of a single peak in order to confirm amplification of a single product and the absence of primer dimers. The optimal quantity of template was assessed using serial dilutions of a control for each gene ranging from 1:1 to 1:10000; the lowest template concentration (1/10000) was acceptable and used for sample analysis. In addition to the three biological replicates for each treatment and time point, each RT-qPCR reaction was carried out in triplicate to capture intra-assay variability. Each assay included three no-template controls (NTC) for each primer pair. Efficiencies were calculated for each gene based on a five-point standard curve using the Cycle Threshold (Ct) value versus the logarithm of each dilution factor and the equation [E = 10^−1/slope^]. All experiments and analyses were conducted following the MIQE guidelines and checklist [27,28].

Two different algorithms were utilized to identify the best reference gene in our experimental design: BestKeeper [29] and NormFinder [30]. Relative expression was determined for each biological replicate and each target gene using the REST tool, which calculates relative expression as the expression ratio (fold change) between Cq values of a target gene versus Cq values for the reference genes [31]. To assess relative expression for the tested genes (GSTs), we firstly determined the best reference gene from the three genes: elongation factor 1α (EFA), 16S and actin. The expression of the GSTs was then normalized and quantified in Log_2_ (experimental/control) as described in Pfaffl et al., [31]. The 1 x-fold expression level was therefore chosen as the threshold for significance of target genes. In one case (EFA, day 0.5, June experiment), one of the three biological replicates was removed as an outlier because the standard deviation (SD) of the mean relative expression of its technical replicates was >1.

### Gene expression using RNA-Seq technology

During the July experiment, high-throughput sequencing was performed on *C*. *finmarchicus* females on days 2 and 5 of the experiment. A total of 18 RNA-Seq libraries (3 replicates × 3 treatments × 2 time points) were sequenced [24]. RNA-Seq reads were quality filtered (FASTX Toolkit, version 0.013; http://hannonlab.cshl.edu/fastx_toolkit/) by trimming the first nine and the last 29 bases, and followed by the elimination of low quality reads (cutoff “Phred” score = 20) as well as Illumina adapters. This resulted in the removal of an average of 34% of reads [24]; reads were then mapped to the *C*. *finmarchicus* reference transcriptome (96,090 contigs) [25] using the software Bowtie (version, 2.0.6) with a 2-nucleotide mismatch tolerance [32]. Identification of significant differences in expression in GST genes was performed using the BioConductor package edgeR [33]. As implemented by edgeR, each library was normalized using the Trimmed mean of M values (TMM) to reduce the differences between library size. Libraries were also normalized using the RPKM method (reads per kilobase of the transcript per million mapped reads); briefly for each gene, the summarized counts were divided by the length of the transcript and the total number of mapped reads in each library using a custom script written in Perl (www.perl.org). Differentially expressed GSTs were statistically identified using the Exact test, implemented by edgeR (parallel to Fisher’s Exact test), based on pairwise comparisons between the control and experimental treatments: CONTROL vs LD and CONTROL vs HD for each time point. In addition, controls at 2 and 5 days were compared statistically to determine whether GST expression changed during the experimental incubation. Transcripts were identified as differentially expressed using the Exact test (p<0.05) and a multiple comparison correction with Benjamini-Hochberg method (false discovery rate <5%) implemented by edgeR [33]. Expression rate was quantified in units of Log_2_ fold (experimental/control) where a value of 0 represents equal expression between the experimental condition and control.

### Comparison between RNA-Seq and RT-qPCR expression levels

Comparison between the RNA-Seq and RT-qPCR measurement was done for all six genes: three glutathione S-transferases (GSTs; 2 cytosolic and one microsomal) and three candidate reference genes (EFA, 16S, actin) genes at days 2 and 5 for the July experiment. RT-qPCR results were compared with the RNA-Seq data in two ways. First, the normalized Cq-values (RT-qPCR) were compared to the normalized counts from RNA-Seq for each gene-treatment-day combination using linear regression. RNA-Seq reads were normalized to counts per kilobase per million mapped reads (RPKM), which normalizes the data by the total number of mapped reads and by the length of each reference transcript. (“kilobase”). Second, for each gene, relative expression as the fold-change difference (Log_2_) between the experimental treatments and controls was compared between the two methods.

## Data Repository

RNA-Seq data are available at the National Center of Biotechnology Information (NCBI; www.ncbi.nlm.nih.gov) under the Bioproject PRJNA312028.

## Results

*Calanus finmarchicus* adult females were fed for 5-days on a control, low dose or high dose diet of *A*. *fundyense*. Survival was high in all experimental jars with mortalities of less then 5% after 5 days. *A*. *fundyense* toxicity remained constant during the 5 day incubation period and was similar in both experiments with an average and standard deviation of 0.020±0.1 ng saxitoxin (STX) eq.cell^−1^ and 0.014±0.5 ng STX eq.cell^−1^ in June and July, respectively [18]. During the July experiment, average ingestion rates were 1μg C female h^−1^ (±0.1 SD) and were similar across all experimental treatments as measured in a parallel grazing study [18]. Further details on the grazing results can be found in Roncalli et al., [18].

### RT-qPCR efficiencies and analysis of candidate reference genes

Amplification efficiencies of the three GSTs and the candidate reference genes ranged between 1.98 and 2.09 (99–110%)(Table 1).

Among the three candidate genes, EFA was found to be the best reference in both June and July experiments: it had the lowest standard deviation (BestKeeper) and the highest stability value (NormFinder) compared with the other two genes (Table 2). Thus, changes in expression for the GSTs and the other two candidate reference genes were normalized using elongation factor 1α (EFA). For the comparison between RT-qPCR and RNA-Seq we used all six genes (see below), and the expression of EFA was normalized using the second best candidate, which according to both metrics was actin (Table 2), following the MIQE guidelines [27,28].

**Table 2:** Potential reference genes assessed with BestKeeper and NormFinder for the June and July experiments.

	EFA	16S	ACTIN
**June experiment**			
*Best Keeper*			
N	36	36	36
Mean	18.94	17.32	25.14
Min	16	14.4	22
Max	23	19.4	28
SD	0.96	0.99	0.98
*NormFinder*			
Stability value	0.71	0.47	0.64
**July experiment**			
*Best Keeper*			
N	27	27	27
Mean	22.98	17.59	28.04
Min	21.09	16	25
Max	24.3	20	29.8
SD	0.7	0.8	0.8
*NormFinder*			
Stability value	0.77	0.46	0.6

Number of replicates (N) includes 3 biological replicates for 3 treatments (CONTROL, LD, HD), 4 time points (0.5, 1, 2,5) for June and 3 time points (0.5,2,5) for the July experiment. The mean, minimum, maximum and standard deviation (SD) of N are reported for BestKeeper and the stability value for NormFinder. The gene with the lowest SD and highest stability value was selected as reference gene.

### Relative gene expression of Glutathione S-transferases using RT-qPCR

**Fig 1:**
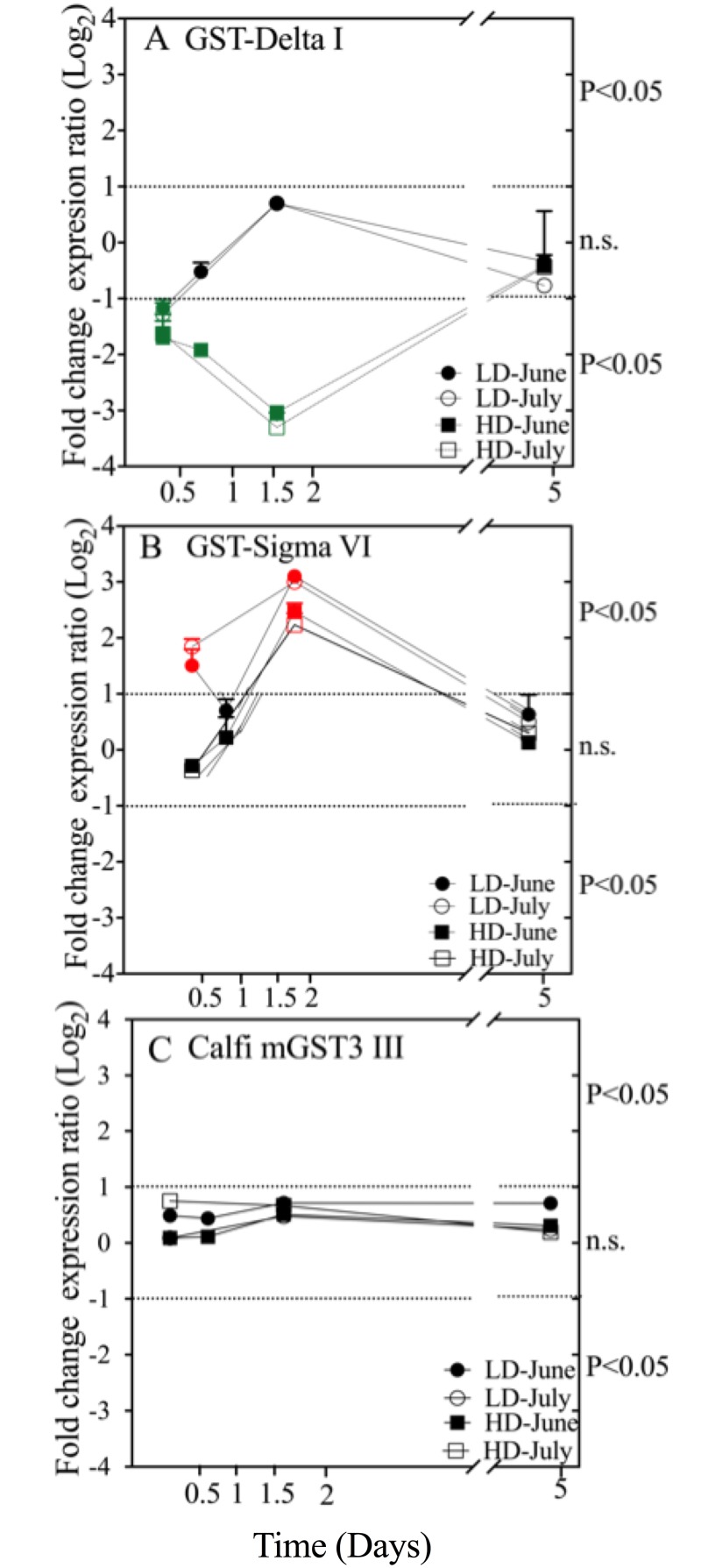
Glutathione S-transferase relative expression over time in *C*. *finmarchicus* feeding on *A*. *fundyense* diets for a total of 5 days. Relative expression of three GSTs members of the cytosolic Delta I (A) and Sigma VI (B) subclasses and the microsomal mGST-3 III (C) was measured at each time point for *C*. *finmarchicus* adult females feeding on LD and HD of *A*. *fundyense* in June and July experiments using RT-qPCR. GST expression (Log_2_ fold change) was normalized using EFA. The dotted lines delimit expression levels that are not significantly different from the control (Log_2_ Fold change < = 1). Solid symbols: June experiment; open symbols: July experiment; circles: LD treatment; squares: HD treatment. Significant differential expression of Calfi Delta I (A) between the control and the experimental treatment is shown in green, and for GST-Sigma VI (B) in red. Day 1 data for July experiment are not available.

The effect of *A*. *fundyense* on the relative expression of two GSTs was time- and dose- dependent (Fig 1). The two cytosolic GSTs (Calfi-Delta I and Calfi-Sigma VI; Fig 1A and 1B) were significantly regulated over time, while the microsomal GST (Calfi-mGST3 III) did not show significant changes in expression compared with the control group at either dose (LD, HD) or in either experiment (June, July) (Fig 1C). Down-regulation of Calfi-Delta I had occurred by 12 hr after the introduction of the toxic dinoflagellate into the diet, and the responses in both LD and HD treatments were similar (Fig 1A). At 24 hours and thereafter, relative expression of this GST in the LD females was not significantly different from the control. In the HD treatment, significant down-regulation of Calfi-Delta I was observed through day 2, but not at day 5. The gene expression patterns observed in both June and July experiments were similar.

Relative expression of Calfi-Sigma VI showed an interesting pattern. In the HD treatment, relative expression of this GST increased between 12 hr and 2 days with significant up-regulation occurring only at 2 days (Fig 1B). In contrast, significant up-regulation of this Sigma GST in the LD treatment occurred at 12 hr and 2 days in both June and July experiments (Fig 1B). Interestingly, at 1 day, relative expression in June in the LD was not significantly different, and similar expression was measured for the HD. This pattern was consistent among all biological replicates in both treatments with coefficient of variation less then 0.2 for both experiments. Unfortunately, there was not an equivalent time point during the July experiment. The magnitude of the differential expression of the Calfi-Delta I and Calfi-Sigma VI was modest and ranged between 2.2- to 3.7-fold change compared with control females feeding on the *Rhodomonas* sp. diet (Fig 1A and 1B).

**Fig 2:**
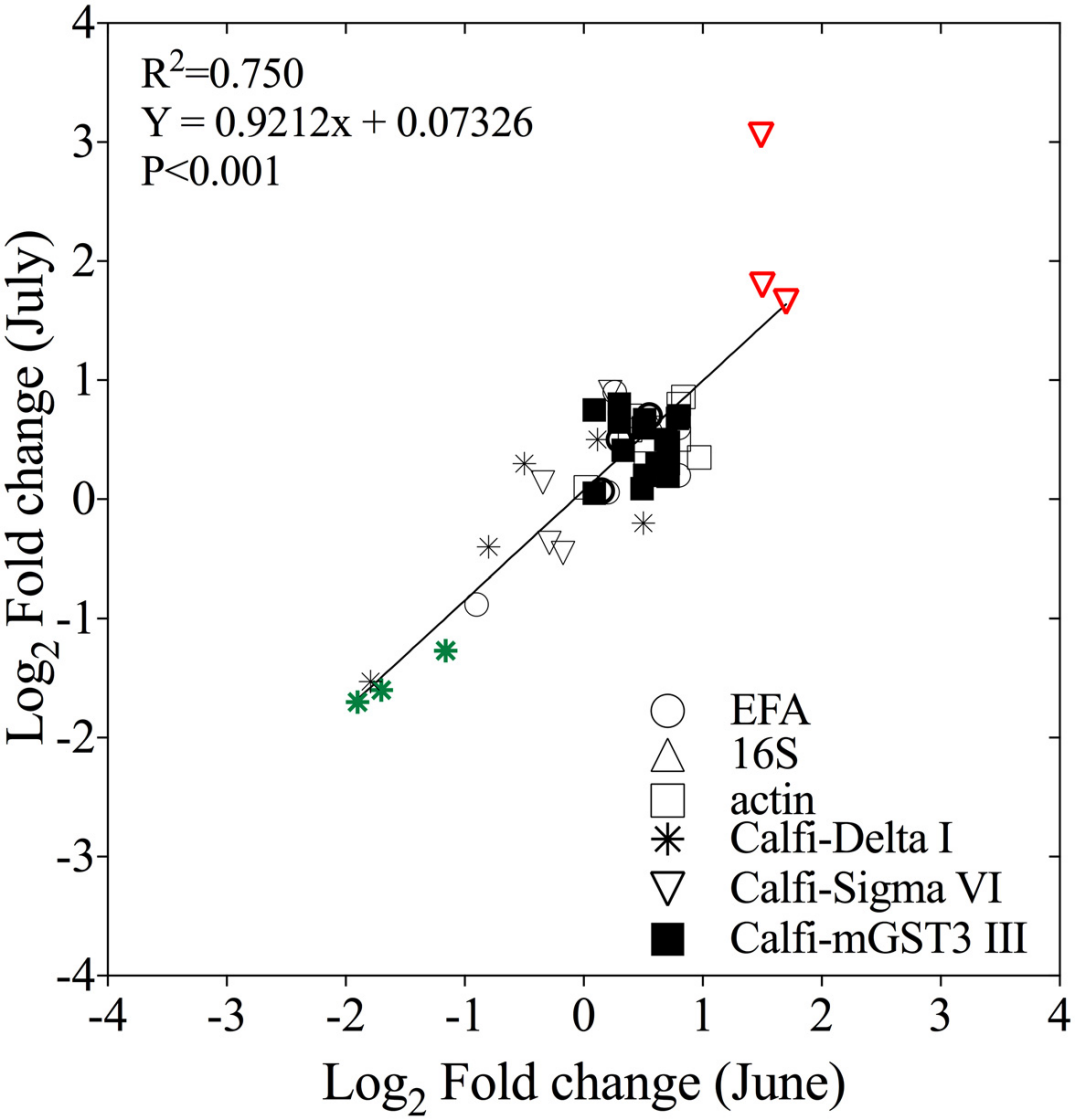
Comparison of relative expression levels measured with RT-qPCR for June and July experiments. The line predicts a 1:1 correspondence (R^2^ = 0.750, P<0.0001). The graph includes expression level (Log_2_ fold change) for 6 genes including 3 glutathione S-transferase (Calfi Delta I, Calfi Sigma VI, Calfi mGST3 III) and 3 candidate reference genes (EFA, 16S, actin) measured in *C*. *finmarchicus* adult females feeding on LD and HD of *A*. *fundyense* for 0.5, 1, 2 and 5 days (n = 36). Expression levels were calculated as the average of 3 biological replicates for each gene, time point and treatment combination. Genes with Log_2_ fold change higher than 1 are considered significantly expressed. In both June and July, Calfi Delta I (in green) and Calfi Sigma VI (in red) were the only genes with significant differential expression compared with the control. Calfi-Delta I was down-regulated exclusively in the HD and Calfi-Sigma IV up in LD and HD treatments. Expression for EFA has been normalized with actin.

One concern in gene expression studies is whether the responses are consistent across experiments, particularly when the experimental individuals are field-collected at different times. Expression levels in the June and July experiments were compared to assess for consistent expression levels across temporally separate experiments. Good agreement was found between the two experiments. Fig 2 shows the fold-change of the difference in expression between the experimental treatments (LD, HD) and controls for the six target genes at 0.5, 2 and 5 days. The fold-change measured in the two experiments was similar as indicated by a highly significant correlation coefficient (R^2^ = 0.750, P<0.0001) and the clustering of the data points around the line that predicts a 1:1 correspondence. For these genes relative expression was very similar in the two experiments, in spite of the differences in reproduction that were observed between the June and July females in all three treatments [18]. The consistency in the response measured in the two experiments (up-regulation of Calfi-Sigma VI, and down-regulation of Calfi-Delta I) adds confidence in the use of relative gene expression as a marker for physiology.

### Comparison between RT-qPCR and RNA-Seq

**Fig 3:**
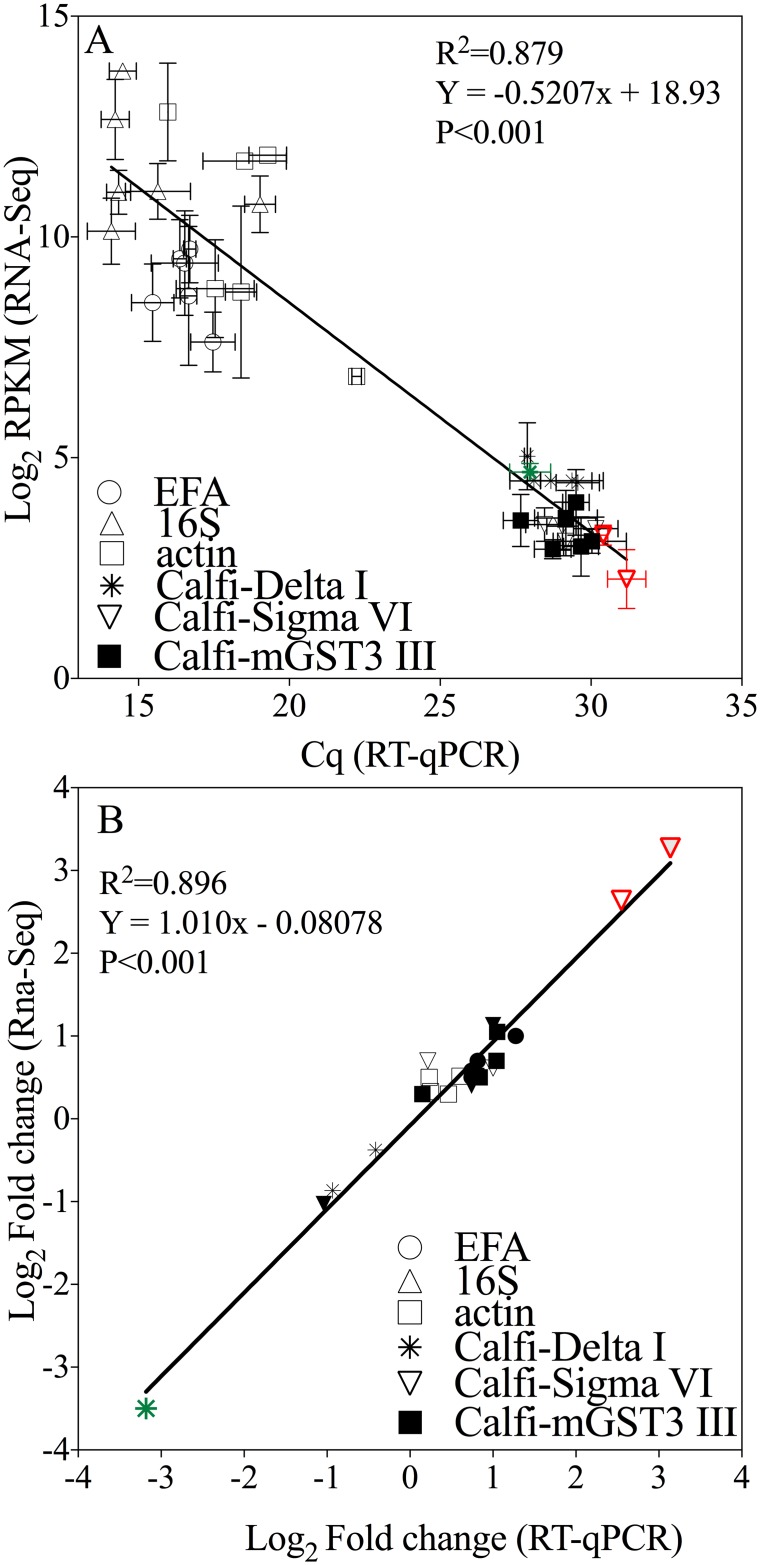
Comparison between relative expression measured with RNA-Seq and RT-qPCR. Relative expression measured with RNA-Seq and RT-qPCR for 6 genes including 3 glutathione S-transferases (Calfi Delta I, Calfi Sigma VI, Calfi mGST3 III) and 3 candidate reference genes (EFA, 16S, actin) in *C*. *finmarchicus* adult females feeding on control *Rhodomonas* sp. or on one of two experimental diets (LD and HD of *A*. *fundyense*) for 2 and 5 days (n = 36). In A) relative expression as reads per kilobase per million mapped reads (RPKM) (Log_2_) obtained from RNA-Seq are compared with Cq-values from RT-qPCR (n = 36). The line is a least-squares linear fit to the data (R^2^ = 0.879, P<0.0001). In B) relative expression as fold change difference (Log_2_) between the experimental and control treatments (n = 24) with genes significantly regulated showing fold-change >1. Calfi-Delta I (in green) and Calfi-Sigma VI (in red) were the only genes showing significant differential expression. Calfi-Delta I was down-regulated exclusively in the HD and Calfi-Sigma VI up in LD and HD treatments. Expression for EFA has been normalized with actin. The line is a least-squares linear fit to the data (R^2^ = 0.896, P<0.0001).

Relative gene expression for all six genes measured by RT-qPCR was compared with RNA-Seq data on days 2 and 5 for the July experiment. In the first comparison relative expression was calculated as Cq-values from the RT-qPCR and RPKM for RNA-Seq runs (Fig 3A). Excellent agreement was found between the two methods with the data fitting through a least-squares that was highly significant (R^2^ = 0.879, P<0.0001; Fig 3A).

The second comparison involved calculating relative expression as fold-change difference (Log_2_) between the experimental treatments and control using relative expression obtained by RT-qPCR and normalized RNA-Seq counts (Fig 3B). The two methods also showed good correspondence (Fig 3B; R^2^ = 0.896, p<0.0001). The relative expression obtained by RNA-Seq confirmed that Calfi-Delta I and Calfi-Sigma VI were differentially expressed (down-regulation and up, respectively) in females feeding on *A*. *fundyense* diets on day 2 but not on day 5 (Fig 3B). Furthermore, magnitude of expression differences was comparable between the two methods and ranged between 2.6 to 3.4 fold (Log_2_). The third GST (Calfi-mGST3 III) was not differentially expressed on either day, or experimental treatment using either method for measuring relative gene expression (Fig 3B).

### Relative Expression of all GSTs in *C*. *finmarchicus*—RNA-Seq

**Fig 4:**
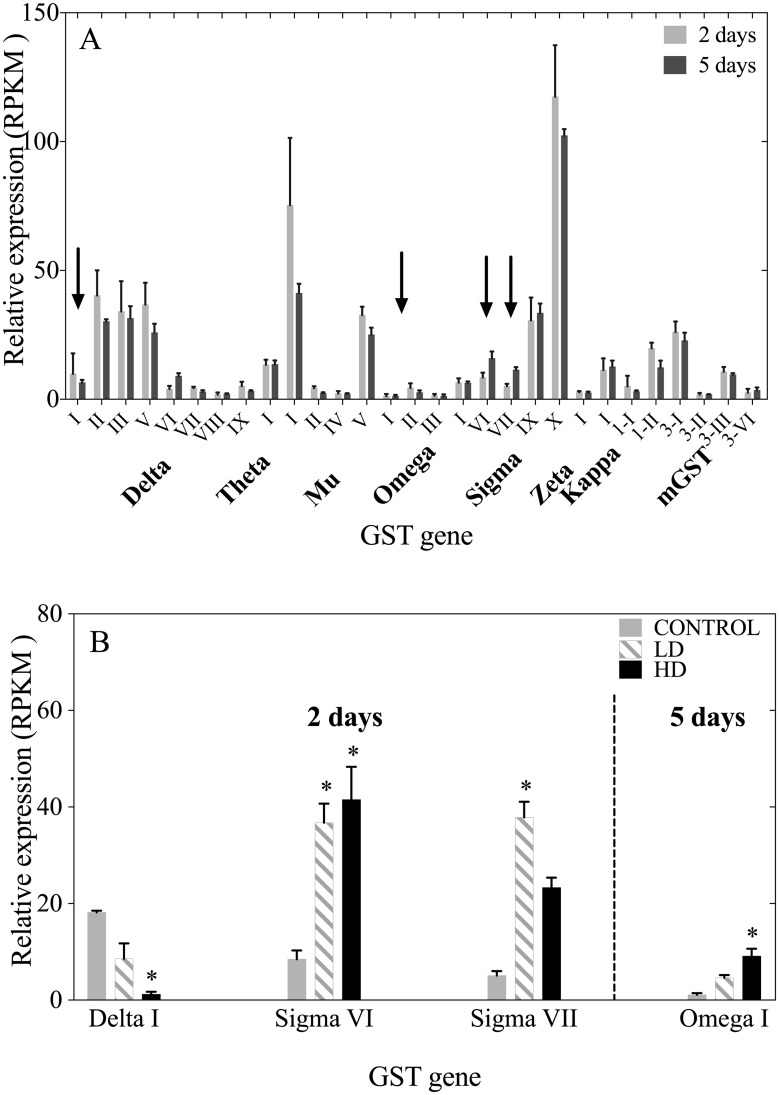
Glutathione S-transferase relative expression in *C*. *finmarchicus* adult females measured with RNA-Seq. A) Relative expression as reads per kilobase per million mapped reads (RPKM) in *C*. *finmarchicus* adult females feeding on the control diet (100% *Rhodomonas* sp.) for 2 and 5 days. GSTs with RPKM <0 (see text n = 8) have been excluded from the graph. Vertical arrows point to differentially expressed GSTs (shown in B). B) GSTs that were differentially regulated in *C*. *finmarchicus* females feeding on either LD or HD of *A*. *fundyense* compared with the control diet for 2 and 5 days. Asterisks indicate the treatment in which the gene was significantly regulated compared with the control diet.

RNA-Seq analysis at 2- and 5-days of *C*. *finmarchicus* females provided expression data for a total of 39 members of the GST family including members of the cytosolic, microsomal and mitochondrial classes. Relative expression varied by GST, but not by time point. Expression levels of all 39 GSTs were similar at the 2 and 5-day time points (Fig 4A). In general, GSTs are expressed at modest levels (2–15 RPKM), as shown for adult females feeding on the control diet at 2 and 5 days (Fig 4A). Six GSTs showed expression levels between 25 and 30 RPKM with highest expression observed in one Sigma GST (Calfi-Sigma X, 2 days: 117 RPKM and 5 days: 92 RPKM) and one Mu GST (Calfi-Mu I, 2 days: 75 RPKM and 5 days: 44 RPKM). None of these eight GSTs were differentially expressed between the control and experimental treatments. Instead, the four GSTs that were differentially expressed were expressed at moderate levels in the controls (< 20 RPKM; Fig 4B). Another eight GSTs were expressed at levels below 1 RPKM in the controls and experimental treatments, and were excluded from the statistical analysis for differential gene expression.

Only four GSTs showed significant differential expression between the control and the experimental treatments (Fig 4B). These GSTs were all members of the cytosolic class. In addition to the Calfi-Delta I and Calfi-Sigma VI (Fig 3), a second Sigma GST (Calfi-Sigma VII) was up-regulated in the LD with a 6-fold increase in its expression compared with the control on day 2, but not on day 5. Expression of the fourth GST, Calfi-Omega I, was highly dose dependent being significantly up-regulated only in the HD treatment with a 10-fold higher expression compared with the control females. This gene was differentially expressed on day 5 but not on day 2 (Fig 4B).

## Discussion

The glutathione S-transferases are a family of proteins involved in a variety of cellular functions, including detoxification [3]. The cytosolic GSTs are involved in Phase II of the detoxification process: they catalyze the conjugation of a toxic/foreign chemical with glutathione in its reduced form (GSH), which in turn increases solubility and facilitates the excretion of the xenobiotic compound [34]. This highly diverse cytosolic class uses variations in one of the two active sites (H-site) to bind to different substrates [35]. Forty-one different GSTs have been identified in *C*. *finmarchicus* in two independently generated *de novo* transcriptomes with 39 of these present in the reference transcriptome used in this study [25]. A large diversity of GST genes have been described in phytophagous insects, such as *Drosophila melanogaster*, *Tribolium castaneum*, *Acyrthosiphon pisum* and *Bombyx mori* [13]. This high gene diversity is thought to be related to the ability of these insects to adapt to a broad range of xenobiotics (e.g. plant chemicals)[3, 36]. Similar to these insects, *C*. *finmarchicus* as a filter-feeder consumes a variety of phytoplankton and microzooplankton, including many that produce toxic secondary metabolites such as diatoms and dinoflagellates [37–39]. If the high diversification of cytosolic GSTs found in *C*. *finmarchicus* is an adaptation for detoxification of xenobiotics as it has been proposed for insects [3,36], then the up-regulation of one or more cytosolic GSTs while the copepod is feeding on *A*. *fundyense* would be predicted. Surprisingly, only two Sigma (VI and VII) and one Omega (I) GST were up in the experimental females, and the response limited to the initial two days for Sigma (VI and VII) and day 5 for Omega I. For Sigma VI, the relative expression results were confirmed in both June and July experimnets and using both RNA-Seq (July) and RT-qPCR.

When insects are exposed to pesticides and/or plant allelochemicals, the Delta and Epsilon GSTs are among the most up GSTs given their specific role in detoxification [40,41]. Although the Epsilon GSTs are insect-specific, 11 Delta GST genes were predicted from the *C*. *finmarchicus* transcriptomes [16]. However, only a single one of these genes (Calfi-Delta I) was differentially regulated, and it was down-regulated raising the question whether the response observed in *C*. *finmarchicus* could be attributed to detoxification of xenobiotic compounds produced by the dinoflagellate. Similarly, in the congener *C*. *helgolandicus* feeding on the toxic dinoflagellate *Karenia brevis* for 5 days, a GST-Delta (most similar to Calfi-Delta IV) was down-regulated with a modest fold-change difference compared with the control diet [9]. In addition, the same GST Delta was not significantly regulated when *C*. *helgolandicus* was exposed for 2 or 5 days to the PUA-producing *Skeletonema marinoi* that had been previously shown to induce differential expression of other detoxification genes [7,8,10]. Overall, these results suggest that in contrast to the insects, GST members of the Delta subclass might not be involved in the copepod detoxification process from natural toxic metabolites present in its diet.

Induction of GST activity in response to xenobiotics is not only typically characterized by the up-regulation of multiple GSTs, but also by the persistence of regulation for multiple days [14,42]. In the copepod *Tigriopus japonicus*, investigation of 5 GSTs (Delta, Sigma, Theta, Omega and mGST1) over 96 hr exposure to heavy metals, showed a significant time-dependent increase for Sigma, Delta and mGST1 with up-regulation, present at 12 hr and persisting over time [14]. In contrast, for *C*. *finmarchicus* although both Sigma and Delta GSTs were rapidly regulated (within 12 hr) after the introduction of the toxic dinoflagellate, their induction did not persist over time. The rapid GST up-regulation and the transient response after the introduction of *A*. *fundyense* is consistent with a general cellular stress response (CSR), a response that is activated when an organism encounters an environmental stressor and involves the regulation of ≥ 1000 genes [43]. The stress response typically includes the up-regulation of some detoxification enzymes (e.g. GSTs), as toxic metabolites produced by the cellular response are removed from the cells [44]. Global gene expression analysis indicated that the introduction of *A*. *fundyense* activated in *C*. *finmarchicus* a cellular stress response at both low and high doses of *A*. *fundyense* already after 2 days [24]. However, this CSR did not involve a significant detoxification activity, with only 8% of *C*. *finmarchicus* detoxification enzymes activated in response to the dinoflagellate [24]. In conclusion, the up-regulation of the GST Sigma observed here is likely to be part of general cellular stress response, since the response disappeared by day 5. However, not enough is known about the specific function of the individual GSTs in *C*. *finmarchicus* or any other crustacean to differentiate between a general stress response vs. a specific response to *A*. *fundyense*.

A similar modest GST response was observed in the cladoceran *Daphnia pulex* feeding on the neurotoxin-producing cyanobacterium *Microcystis aeruginosa* [45]. Among the 12 investigated cytosolic GSTs (Delta, Sigma, Zeta and Mu), a single Zeta GST was differentially regulated in response to the stressor; this gene was up with a modest 2-fold difference in expression compared with the control. These authors also concluded that detoxification was not a major component of the physiological response of *D*. *pulex* feeding on *M*. *aeruginosa* [45].

The combination of RNA-Seq and target gene approach here provided data on relative expression of 39 GSTs for two time points in one experiment, while RT-qPCR for the target GSTs added to these data by providing additional time points, as well as gene expression data from two independent experiments. Overall, the good agreement found here between RT-qPCR and RNA-Seq data, and the modest expression levels (5–10 RPKM) for most of the genes, confirms a high level of sensitivity of detection and consistency between platforms. These results are consistent with other studies that have found good agreement between RT-qPCR and RNA-Seq [46,47].

Comparable results were obtained for the two experiments, in spite of differences between females collected in June and July from the Gulf of Maine. Experiments on female reproductive success were conducted in parallel to the gene expression studies, and these suggest that the June and July females differed in their reproductive potential with smaller brood sizes in the June control females compared with the July ones [16]. Nevertheless, the response at the transcriptional level measured here as relative gene expression of the three target GSTs was very similar, with significant differential expression occurring at both low and high dose.

## Conclusions

Two independent methods (RT-qPCR and RNA-Seq) were used to examine changes in the expression of GST genes in *C*. *finmarchicus* in response to the dinoflagellate *A*. *fundyense*. Results from RNA-Seq and RT-qPCR platforms were in agreement for both GSTs and reference genes. Females showed an initial and transient response that included the up-regulation of two Sigma (VI and VII) GSTs and down-regulation of a single Delta (I) GST. Based on RNA-Seq data, a single GST (Omega I) was up on day 5. Changes in relative expression were already present at the first time point (0.5 day), underscoring that there was an immediate response to the introduction of the dinoflagellate diet. Relative gene expression patterns in females from the June and July experiments gave very similar results, in spite of differences in overall reproductive rates. Although detoxification is likely to be part of the copepod’s response to the dinoflagellate diet, it does not appear to be the major response, given the modest changes in gene expression as measured by the number of regulated GSTs (4 out of 39 in the reference transcriptome) and the magnitude of the differential expression (< 4-fold difference).
